# A detailed report of the resource use and costs associated with implementation of a short stay programme for breast cancer surgery

**DOI:** 10.1186/s13012-015-0270-9

**Published:** 2015-05-27

**Authors:** Stephanie M.C. Ament, Mascha de Kok, Cornelis J.H. van de Velde, Jan A. Roukema, Toine V.R.J. Bell, Fred W. van der Ent, Trudy van der Weijden, Maarten F. von Meyenfeldt, Carmen D. Dirksen

**Affiliations:** Department of Family Medicine, School for Public Health and Primary Care (CAPHRI), Maastricht University Medical Centre, P.O. box 616, 6200 MD Maastricht, The Netherlands; Department of Clinical Epidemiology and Medical Technology Assessment (KEMTA), Maastricht University Medical Centre, P.O. box 5800, 6202 AZ Maastricht, The Netherlands; General Practice centre “Het Anker”, Seringenstraat 259, 3142 NV Maassluis, The Netherlands; Department of Surgery, Leiden University Medical Centre, P.O. box 9600, 2033 RC Leiden, The Netherlands; Department of Surgery, St. Elisabeth Hospital, P.O. box 90151, 5000 LC Tilburg, The Netherlands; Department of Surgery, Laurentius Hospital, P.O. box 920, 6040 AX Roermond, The Netherlands; Department of Surgery, Orbis Medical Centre, P.O. box 5500, 6130 MB Sittard-Geleen, The Netherlands; Department of Surgery, Maastricht University Medical Centre, P.O. box 5800, 6202 AZ Maastricht, The Netherlands

## Abstract

**Background:**

Despite the increased attention for assessing the effectiveness of implementation strategies, most implementation studies provide little or no information on its associated costs. The focus of the current study was to provide a detailed report of the resource use and costs associated with implementation of a short stay programme for breast cancer surgery in four Dutch hospitals.

**Methods:**

The analysis was performed alongside a multi-centre implementation study. The process of identification, measurement and valuation of the implementation activities was based on recommendations for the design, analysis and reporting of health technology assessments. A scoring form was developed to prospectively determine the implementation activities at professional and implementation expert level. A time horizon of 5 years was used to calculate the implementation costs per patient.

**Results:**

Identified activities were consisted of development and execution of the implementation strategy during the implementation project. Total implementation costs over the four hospitals were €83.293. Mean implementation costs, calculated for 660 patients treated over a period of 5 years, were €25 per patient. Subgroup analyses showed that the implementation costs ranged from €3.942 to €32.000 on hospital level. From a local hospital perspective, overall implementation costs were €21 per patient, after exclusion of the costs made by the expert centre.

**Conclusions:**

We provided a detailed case description of how implementation costs can be determined. Notable differences in implementation costs between hospitals were observed.

**Trial registration:**

ISRCTN: ISRCTN77253391

**Electronic supplementary material:**

The online version of this article (doi:10.1186/s13012-015-0270-9) contains supplementary material, which is available to authorized users.

## Background

Innovations often do not translate automatically into routine care [1]. Therefore, investment in active implementation is generally needed to introduce and embed changes in health care practice [2, 3]. Implementation is a complex process and there are no magic bullets for optimal implementation success [4, 5]. The value of implementation strategies depends on both the degree of change achieved following implementation and on the efforts and costs associated with implementation. Implementation costs can be made during the innovation development, implementation strategy development and the execution of the implementation strategy [6]. Not including costs and effects related to implementation of an innovation may produce biased and overestimated cost-effectiveness results [7]. Also, costs of the health care process itself may have changed. Whereas the number of reports on effectiveness of implementation activities is increasing [8], most implementation studies do not report implementation costs. Even if implementation costs are reported, details as regards to the methods used for the identification, measurement and valuation of the costs are mostly lacking [9–12]. Providing such details will offer some methodological guidance, which may lead to more robust and generalisable study findings. Also, accurate information on activities and costs associated with implementation of an innovation is important for policymakers such as hospital managers, especially regarding institutions where implementation of an innovation has not yet taken place.

Between 2004 and 2007, a study was performed regarding multi-centre implementation of a short stay programme for breast cancer surgery (SSP) [13]. Short stay was defined as admission, surgery and discharge the same day (day-case admission) or discharge within 24 h after surgery (also referred to as overnight stay). The SSP consisted of 29 recommendations and was aimed at improving patient education, providing more standardised and better organised care, reducing hospital stay, and high quality of care as assessed by patients. Results of the economic evaluation comparing SSP with care as usual revealed that the intervention SSP was more cost-effective, both from a societal and a health care point of view, mainly due to a substantial cost reduction [14, 15].

In the current study, we provide a detailed report of the resource use and costs associated with a hospital-tailored strategy to implement SSP in four early adopter hospitals.

## Methods

### Design

In the present prospective observational study, an implementation cost-analysis was performed. This study was executed alongside a multi-centre before-after implementation study, from December 2004 until December 2007 in the Netherlands. The protocol of the implementation study has been published elsewhere [13].

### Implementation costing method

The implementation costing method focused on the prospective identification, measurement and valuation of implementation activities. Revealed implementation costs were based on the investments (e.g. time, materials) made by the hospitals, for which they did not receive a financial compensation. The process of identification, measurement and valuation of the implementation activities was based on recommendations for the design, analysis and reporting of health technology assessments [16, 17]. Drummond and co-workers described these recommendations to improve the reporting and the generalisability of economic evaluations. In the current study, implementation costs were determined from the hospitals’ perspective and from the perspective of the external implementation team.

To facilitate prospective micro-costing in measurement of the implementation costs, a scoring form was developed to identify and measure activities referring to personnel costs and material costs (e.g. travel costs and institutional incentives) dedicated to implementation activities (Additional file 1). The scoring form had to be filled out individually by each health care professional participating in plenary local team meetings in each of the hospitals and by experts of the Maastricht University Medical Centre (MUMC). To determine the content, the intensity and duration of the activities on an individual level, the name and position of the person who filled out the form, the date of activities, the time spent on the activity, a description of the activities (e.g. the forms also addressed time spent on writing emails to the researcher) and the names and positions of the other attendees were recorded. The forms were collected by the researcher during the plenary meetings or, if a person was not able to attend these, the forms were sent by mail to the researcher. All activities scored by the attendees were checked by the researcher and the cost-effectiveness expert to prevent miscalculations. A time horizon that captured the expected consequences [18] of the implementation strategy was used to determine the mean implementation costs per patient. This time horizon was set at 5 years. Implementation costs were calculated by multiplying resource use with a cost-price per unit of resource use. Health care professionals’ time-related costs were calculated based on the staff member’s gross salary per hour [19], including 38 % employers’ social charges and 10 % housing costs (i.e. depreciation and maintenance of buildings and apparatus) multiplied by the number of hours. Costs were expressed as 2013 Euros (€).

### Study population and setting

The study was performed in four Dutch hospitals recognised as early adopter hospitals regarding breast cancer care. Early adopter hospitals are active in changing and innovating health care and function as a reference group for most innovators [20]. The participating hospitals were a university hospital, a large training hospital, a small training hospital and a non-training hospital.

### The implementation strategy

The specific strategies will be discussed in the results, but the generic aspects are that the implementation strategy was targeted at the professionals involved in the breast cancer surgery process and was externally guided by an expert team from the MUMC. The expert team consisted of a researcher, an implementation expert, a cost-effectiveness expert, a statistician and the project leader (oncologic surgeon). The strategy was hospital-tailored as it was executed according to the model of effective implementation by Grol and Wensing to address the specific circumstances in each of the participating hospitals [1, 21]. The implementation strategy was developed and executed over a time period of 36 months.

### Data analyses

Implementation activities were categorised in implementation phases: development of the strategy to implement SSP (developmental costs) and costs for execution of the strategy (execution costs) [6]. As the innovation (i.e. the SSP guidelines) was developed at the expert centre, one-time costs related to the development of the innovation before implementation were not included [18]. Research-related costs (e.g. time spent on writing scientific papers on the implementation study) were excluded from the analysis. Subgroup analyses of implementation costs were performed on hospital level and from a hospital perspective after exclusion of the costs made by the expert centre.

## Results

### Study population

Between December 2004 and December 2007, hospital-tailored strategies were developed and executed in the four participating hospitals. Surgeons, breast nurses, nurse practitioners, department heads, department nurses, team leaders and home care nurses were represented in most of the executed activities. Participants who were present in lower frequency were staff advisors, research nurses, division managers, financial advisors, anaesthesiologists, anaesthesiology nurses, liaison nurses, secretaries and social workers. Table 1 displays which disciplines were involved in each hospital.

**Table 1:** Involvement of the disciplines on hospital level during implementation

Function	Hospital 1	Hospital 2	Hospital 3	Hospital 4
Nurse practitioner	No	Yes	No	Yes
Breast care nurse	Yes	Yes	Yes	Yes
Surgeon	Yes	Yes	Yes	Yes
Radiologist	No	No	No	Yes
Team leader	Yes	Yes	Yes	Yes
Staff advisor	yes	Yes	No	No
Ward manager	yes	Yes	No	Yes
Division manager	yes	Yes	No	No
Financial advisor	yes	No	No	No
Anaesthesiologist	yes	Yes	No	No
Anaesthesiology nurse	Yes	Yes	No	No
Secretary	yes	Yes	No	No
Social worker	No	Yes	No	No
Admission planner	yes	No	No	yes
Nuclear specialist	Yes	No	No	No
Senior nurse	Yes	Yes	Yes	Yes
Junior nurse	Yes	Yes	Yes	Yes
Liaison nurse	Yes	Yes	No	Yes
Patient support member	No	No	No	Yes

### Implementation activities

Different types of implementation activities were conducted during the implementation project. A multi-faceted implementation strategy was developed and executed in each participating hospital, tailored to the hospital’s specific needs and guided by the expert team. Table 2 shows the executed activities and the duration of the activities on hospital level. Strategy development related meetings were organised at the expert centre. During the expert meetings at the expert centre, important issues were discussed: steps and decisions taken previously by the researcher, the exploration of barriers and facilitators as part of the problem analysis for implementation and the implementation strategy. Main achievements were preparation and summarisation of the implementation strategy, the latter serving as reminders concerning agreements that had been made and assignments regarding implementation activities for each participant in the meeting (e.g. the breast nurse developing flyers and the surgeon corresponding with hospital managers). For the organisation and content of the meetings, the researcher was supported by the expert team. Before the start of the implementation strategy, educational meetings were organised at each hospital to provide the local project leaders information about SSP.

**Table 2:** Summary of hospital-tailored implementation strategy activities and costs to implement a short stay programme for breast cancer surgery

	Total	Hospital 1		Hospital 2		Hospital 3		Hospital 4		Expert centre	
Type of implementation costs	Costs (€)	*n* (hours)	Costs (€)	*n* (hours)	Costs (€)	*n* (hours)	Costs (€)	*n* (hours)	Costs (€)	*n* (hours)	Costs (€)
Developmental costs											
Expert meeting											
Expert meeting at the expert centre	1.307	N.A	N.A	N.A	N.A	N.A	N.A	N.A	N.A	8	1.307
Educational meeting											
Information provision to local project leaders	2.072	5	518	5	518	5	518	5	518	N.A	N.A
Subtotal	3.379		518		518		518		518		1.307
Developmental costs/execution costs											
Multidisciplinary meeting											
Plenary multidisciplinary team meetings	37.699	55.1	16.580	22.1	7.010	2	1.074	28.3	7.367	105	5.669
Small meetings (average 2.9 participants)	11.520	26.9	5.463	6.4	1.161	8.3	1.842	10.5	2.707	N.A	N.A
Meetings to adapt the guideline to the local level	9.923	25.3	2.418	50.3	4.087	0	0	23.7	3.287	1	131
Communication and media											
Telephone conference	578	3.0	403	1.6	175	0	0	0	0	N.A	N.A
E-mail conference	290	0.7	55	3.5	235	0	0	0	0	N.A	N.A
Development of instruction leaflet	255	0.5	23	4.3	231	0	0	0	0	N.A	N.A
Travel costs	7.143	N.A	120	0	0	0	0	0	0	N.A	7.023
Subtotal	60.265		25.063		12.899		2.916		13.360		12.823
Execution costs											
Education											
Training staff at the expert centre	847	0	0	1	54	0	0	8	793	N.A	N.A
Clinical lectures given by the breast nurse from the expert centre	11.464	40	6.314	7.5	2.321	1	508	9.0	2.320	1.8	347
Incentive											
Institution incentive	195	0.1	105	0	0	0	0	0.1	90	N.A	N.A
Subtotal	12.507		6.419		2375		508		3.204		347
Total	83.293		32.000		15.792		3.942		17.082		14.477

Gross salary per hour (excluding social charges and housing costs): junior nurse, secretary, admission planner: €27,–; breast nurse, home care nurse, member patient support group: €30,–; anaesthetic nurse, senior nurse, social worker: €31,–; nurse practitioner, team leader, ward manager, phD student: €35−; staff advisor: €44,–; ICT expert: €47,–; financial advisor, implementation expert, cost-effectiveness expert, statistician: €51,–; division manager: €60,–; surgeon, anesthesiologist, radiologist, nuclear specialist: €69,–; project leader: €79,–. Costs are expressed in 2013 Euros

During the execution of the implementation strategy, outreach visits were performed by a researcher with the local team (plenary multidisciplinary team meetings) or with the local breast nurses. In the first plenary local team meeting, SSP was explained and discussed. Subsequent meetings were used to identify barriers and facilitators for the implementation of SSP during the project, to tailor the implementation strategy to the needs and wishes of each hospital, to discuss problems that had arisen and to provide feedback on current performance; professionals learned to what extent they adhered to the SSP recommendations and how patients perceived quality of care. Minutes were made of each plenary local team meeting. Clinical lectures were given by an experienced breast nurse from the expert centre to the home care nursing teams and to nurses on the ward. Teams that achieved good performance and needed some motivation received an incentive, for example, snacks during a coffee break. Overall, preparation of project meetings with experts and small-scale meetings were the most frequently performed activities. Participants from all hospitals accounted for a total of 450 h spent on plenary multidisciplinary team meetings.

### Implementation costs

**Fig. 1:**
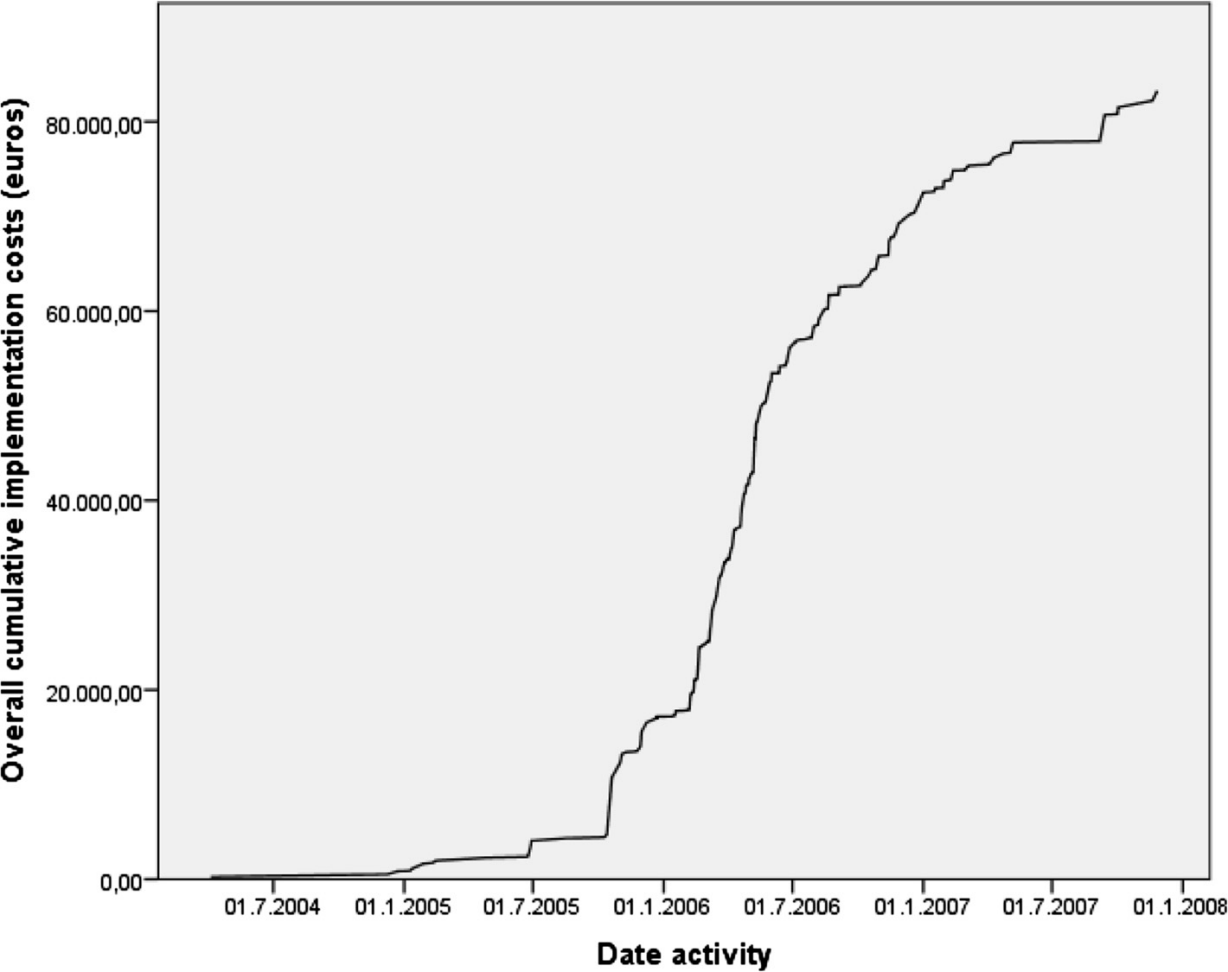
Overall cumulative implementation costs over time

Total implementation costs in the four hospitals were €83.293. In Table 2, the costs of the implementation strategy are presented as total costs and as costs on hospital level. The three largest cost components of the total implementation costs were plenary multidisciplinary team meetings, small meetings (on average consisting of less than three persons) and clinical lectures. Figure 1 shows the cumulative implementation costs of the hospital-tailored strategy over time. Costs in 2007 were mainly related to plenary multidisciplinary team meetings and making the minutes of these meetings. Table 2 shows that 660 patients are treated in the four participating hospitals yearly. Using a time horizon of 5 years, 3.300 (5 × 660) patients are treated in the four early adopter hospitals. Mean implementation costs assessed for 660 patients treated per year with a time horizon of 5 years were €25 (€83.293/3.300) per patient.

### Subgroup analyses

As the implementation strategy was tailored to local needs, differences in the type and in the intensity of implementation activities were shown on hospital level. Hospital 1 showed the highest level of development and execution activities, and in hospital 3, the smallest number of activities was needed to implement SSP. Hospitals 2 showed a relatively high level of communication by e-mail, whereas for hospital 1 and 3, more small-scale face-to-face meetings were executed. On hospital level, the implementation costs ranged from €3.942 in hospital 3 to €32.000 in hospital 1. From a local hospital perspective, implementation costs were €21 per patient in a time horizon of 5 years (€68.816/3.300), after exclusion of the costs made by the expert centre (€83.293 − €14.477).

## Discussion

In the current study, we determined the resource use and costs associated with the implementation of SSP in four early adopter hospitals, using a structured methodology for identification, measurement and valuation of activities. The innovation was implemented by means of a hospital-tailored strategy which was externally guided by an expert team. Most costs were caused by project meetings with implementation experts, clinical lectures and small-scale meetings. This study showed that the overall mean implementation costs were €83.293. Implementation costs were €25 per patient when treating 660 patients a year and using a time horizon of 5 years post-implementation.

Activities related to a change in health care provision were not included in the current study as this part of costs was related to the health care innovation itself and was not part of the implementation strategy. These costs were included in an economic evaluation of SSP versus usual care [15]. The calculated costs in this paper reflect the efforts and time investments by the hospitals and the expert team, for which they were not compensated, and therefore represent true opportunity costs.

Implementation is a complex process and there is no standard strategy for reaching optimal implementation success against the lowest possible costs. As a consequence, implementation costs may vary per implementation project and on institutional level. The study shows that implementation costs varied widely between hospitals, which can be expected given the fact that the implementation strategy was tailored to the hospitals’ needs and circumstances. Notably, an association seemed to be present between the uptake of short stay and total implementation costs per hospital. After finishing the hospital-tailored implementation strategies, in hospital 3, the uptake of the proportion of patients treated in short stay after breast cancer surgery somewhat decreased (−7 %) compared to the preimplementation measurement [15] and implementation costs were €3.942. In hospital 2, the uptake was 37 % and implementation costs were €15.792, in hospital 4 the uptake was 52 % and implementation costs were €17.082, and in hospital 1, the uptake was 71 % and implementation costs were €32.000. It is unknown whether or not the proportions of patients treated in short stay would have been higher if other or more implementation activities had been performed. In hospital 1, health care professionals showed more need of guidance by the expert team than the other hospitals. Although the implementation costs in hospital 1 were twice as high compared with hospital 2, the uptake of short stay was also almost twice as high. Implementation uptake and costs in hospital 3 showed to be lower compared to other hospitals as this hospital started a self-initiated implementation before the start of the official project [15]. The current study provided detailed insight in total implementation costs, as well as implementation costs per hospital and per patient. However, due to an increased breast cancer incidence rate [22], more patients may have been treated in the four hospitals. Treating more breast cancer patients affects the implementation costs on patient level. Therefore, implementation costs on patient level may be overestimated.

We believe that the implementation activities collected by the health care professionals were a true representation of reality, as many activities had been attended by different health care professionals and there was a high agreement in scoring of activities between health care professionals. A weakness of the current study may be that the period of measurement of implementation activities was restricted to the duration of the implementation project. It is likely that more activities have been performed before or after the formal implementation project. For example, the study was performed in four hospitals recognised as early adopter and between approval for study participation and actual start of the study; some professionals from hospital 3 had already visited the expert centre to learn about SSP. The early adopter status was strengthened by the observation that the uptake of short stay seemed to have started already during the care as usual period in one hospital, i.e. before the beginning of externally guided implementation project [15]. As the implementation project was not formally started at that time, the implementation activities associated with this early adoption were not measured prospectively and were therefore not included in the current study. Consequently, it can be argued that the implementation costs are underestimated. To identify and measure all implementation costs, it could be recommended in future research to keep a structured implementation cost log book on hospital level to track implementation activities prospectively before, during and after the formal implementation project. As the activities were not specified on the scoring form, activities may have been missed as they were not perceived as implementation related. On the other hand, costs may be overestimated in case an activity was also performed for other purposes than implementation. It is therefore also recommended in future research to develop a (digital) implementation log book with prespecified implementation activities to be scored on a weekly basis. This form needs to be tested as part of a pilot.

### Recent developments

The current study presented a detailed description of the identification, measurement and valuation of costs by using methodological guidance [10], which enables comparability and reproducibility of the executed strategy by providing detailed and structured information [11].

Recently, the Effective Practice and Organisation of Care (EPOC) Data Collection Checklist intervention categories, the behaviour change wheel, Leeman taxonomy and behaviour change techniques were mapped together in order to develop a more uniform taxonomy and to generate a common implementation activity terminology [23]. This new construction is in its infancy and is a starting point for a new framework aimed at mapping implementation activities. Also, Powell and colleagues [24] recently published a refined compilation of implementation strategies to improve conceptual clarity. These conceptualisations of implementation activities can be used in future research to determine implementation costs. Moreover, the findings of this study suggest that in case of a hospital-tailored implementation strategy, implementation costs need to be determined at an institutional level.

## Conclusions

This study involved a cost-analysis performed using generally accepted principles on implementation of innovations and cost calculations. We provided a case description of how implementation costs can be determined prospectively using a structured and transparent approach to identification, measurement and valuation. The current study may offer methodological guidance to future implementation cost studies which may facilitate the generalisability of implementation study findings.

## Additional file

Additional file 1:
**Implementation activities scoring form.** This file contains the implementation activity scoring form that was developed and used in the current study.

## Abbreviations

EPOC: Effective Practice and Organisation of Care
MUMC: Maastricht University Medical Centre
SSP: short stay programme for breast cancer surgery
