# Supplementary material for: A detailed report of the resource use and costs associated with implementation of a short stay programme for breast cancer surgery
Source: Implement Sci. 2015 May 27;10:78. doi: 10.1186/s13012-015-0270-9 (PMC4449601; doi:10.1186/s13012-015-0270-9)
Supplement: Additional file 1: — Implementation activities scoring form. This file contains the implementation activity scoring form that was developed and used in the current study. [file 13012_2015_270_MOESM1_ESM.doc]

| Date | **Duration (minutes)** | Description activities | Persons present |
| --- | --- | --- | --- |
|  |  |  |  |
|  |  |  |  |
|  |  |  |  |
|  |  |  |  |
|  |  |  |  |
|  |  |  |  |
|  |  |  |  |
|  |  |  |  |
|  |  |  |  |
|  |  |  |  |
|  |  |  |  |
|  |  |  |  |
|  |  |  |  |
|  |  |  |  |
|  |  |  |  |
|  |  |  |  |
|  |  |  |  |
|  |  |  |  |
|  |  |  |  |
|  |  |  |  |
|  |  |  |  |
|  |  |  |  |
|  |  |  |  |
|  |  |  |  |
|  |  |  |  |
|  |  |  |  |
|  |  |  |  |
|  |  |  |  |
|  |  |  |  |
|  |  |  |  |
|  |  |  |  |
|  |  |  |  |
|  |  |  |  |
|  |  |  |  |
|  |  |  |  |

**Centre**

**Name:**

**Function:**
